# Heart rate monitoring reveals differential seasonal energetic trade-offs in male noctule bats

**DOI:** 10.1098/rspb.2024.0855

**Published:** 2024-07-10

**Authors:** Lara Keicher, J. Ryan Shipley, Melina T. Dietzer, Martin Wikelski, Dina K. N. Dechmann

**Affiliations:** ^1^ Max Planck Institute of Animal Behavior, Am Obstberg 1, Radolfzell 78315, Germany; ^2^ Department of Biology, University of Konstanz, Universitätsstraße 10, Konstanz 78457, Germany; ^3^ WLS Institute for Snow and Avalanche Research, Flüelastraße 11, Davos CH-7260, Switzerland; ^4^ Department of Wildlife Ecology and Management, Uni­ver­sity of Freiburg, Tennenbacher Straße 4, Freiburg 79106, Germany; ^5^ Cluster for the Advanced Study of Collective Behaviour, University of Konstanz, Universitätsstraße 10, Konstanz 78457, Germany

**Keywords:** heart rate, energy expenditure, *Nyctalus noctula*, reproduction, spermatogenesis, torpor

## Abstract

Understanding how animals meet their daily energy requirements is critical in our rapidly changing world. Small organisms with high metabolic rates can conserve stored energy when food availability is low or increase energy intake when energetic requirements are high, but how they balance this in the wild remains largely unknown. Using miniaturized heart rate transmitters, we continuously quantified energy expenditure, torpor use and foraging behaviour of free-ranging male bats (*Nyctalus noctula*) in spring and summer. In spring, bats used torpor extensively, characterized by lowered heart rates and consequently low energy expenditures. In contrast, in summer, bats consistently avoided torpor, even though they could have used this low-energy mode. As a consequence, daytime heart rates in summer were three times as high compared with the heart rates in spring. Daily energy use increased by 42% during summer, despite lower thermogenesis costs at higher ambient temperatures. Likely, as a consequence, bats nearly doubled their foraging duration. Overall, our results indicate that summer torpor avoidance, beneficial for sperm production and self-maintenance, comes with a high energetic cost. The ability to identify and monitor such vulnerable energetic life-history stages is particularly important to predict how species will deal with increasing temperatures and changes in their resource landscapes.

## 1. Introduction

Animals need energy to survive, grow, move, and reproduce [1], and meeting these daily energy requirements can be challenging. Small changes in environmental conditions can have large effects on fitness [2,3], especially in animals that depend on ephemeral resources [4]. To deal with adverse conditions, animals have evolved manifold and crucial energy-saving strategies [5,6]. However, how free-ranging animals deal with the often conflicting challenges of their physiological needs and fluctuating changes in their environment is widely unknown. These trade-offs remain critical for understanding not only the evolution of life histories and energy-managing strategies, but also the future consequences of rapidly changing climates.

Bats exemplify the delicate balancing act between energy budgets and compensating strategies. Their energetically expensive mode of locomotion [7], associated high mass-specific metabolic rates [8], and limited capacity to store fat [9] in combination with often unpredictable food availability [10,11], likely promoted strategic torpor use throughout the non-hibernating period in all temperate-zone bats [12]. Torpor is a controlled reduction of metabolic rate and heart rate that is often accompanied by a reduction in body temperature [13]. Torpor is thought to be largely incompatible with periods of rapid somatic growth, and is often avoided during reproductive periods such as pregnancy, lactation and spermatogenesis [14]. The literature often emphasizes the high cost of reproduction for female bats [15–17]. However, during spermatogenesis, testes size can increase to up to 8% of the bat’s body mass [18], and a substantial amount of energy and nutrients are needed to grow tissue and to proliferate germ cells [19,20]. The life-history stage of reproduction adds substantial energetic costs for both sexes, when high body temperatures need to be maintained [21–27]. Bats likely have to balance this by increasing foraging duration and energy uptake [22,28–31]. Indeed, the reproductive cycle of temperate-zone bats seems to correspond directly to both season and food availability [32]. However, in males, as in females, detailed knowledge on how bats balance their dynamic energy budgets as seasonal temperatures, food availability and their reproductive status change is lacking.

Furthermore, at least four species of tropical and temperate-zone bats can have flexibly varying heart rates and a reduced metabolism largely independent of body temperature, providing another dimension to torpor and energy conservation [27,33–36]. In fact, heart rate is a more precise measure of bat energy expenditures than body temperature [27,37]. Due to a lack of suitable miniaturized technology, past studies were largely restricted to the use of transmitters tracking bat skin temperature [38], and any hidden strategies involving a lowered heart rate and metabolic rate remained undetected [27,37].

To understand and accurately quantify energy budgets of bats in different stages of their life cycle, we studied free-ranging male common noctule bats, *Nyctalus noctula*, in southern Germany. *Nyctalus noctula* is a representative insectivorous temperate-zone bat species with an annual life cycle characterized by periods of torpor and hibernation that are timed around different stages of the annual reproductive cycle and seasonal insect availability [14]. *Nyctalus noctula* is an opportunistic generalist and forages on a variety of insect species, including a large proportion of ephemeral insect swarms, especially in spring [39–43]. After mating in late summer and autumn, both sexes use torpor regularly and forage extensively in preparation for hibernation [44]. During hibernation, females store sperm in their uterus until fertilization soon after spring arousal in mid/end of March [18,45,46]. In spring, both sexes again regularly use daily torpor [44], and females migrate northeast to give birth and raise their young during summer, while males from our study population stay in the wintering habitat year-round [43,47]. After female departure in spring, males gather in small male-only ‘bachelor colonies’ of around 20 individuals [44] and start spermatogenesis around June [18,32,48,49]. In this period, torpor use should be reduced in order to complete spermatogenesis before onset of the mating period once the females return [18,50,51]. As soon as spermatogenesis is completed, usually around late July, sperm is stored in the *caudae epididymis*, and males use torpor regularly again [18]. At this time, bachelor colonies dissolve, and males start to establish harem roosts, which are joined by up to 20 females [44,49,52].

We studied free-ranging males tagged with heart rate transmitters in the cool spring (post hibernation) and in the warmer summer when the females had migrated away (during ongoing sperm production) to record and calculate individual energy budgets. This allowed us to investigate how individuals seasonally allocated time and energy to different activities such as flight, rest and torpor. We predicted that in spring, male bats would spend most of their time in torpor, avoiding the costs of thermoregulation. This should be reflected by low heart rates and result in low overall energy expenditures. In summer, when male bats produce sperm, we predicted reduced use of torpor, reflected by higher heart rates and higher overall energy expenditures. Even though higher ambient temperatures lower thermoregulatory costs in the summer compared with the thermoregulatory costs in the spring, overall summer energy expenditures will exceed those of the spring. Assuming higher insect abundance in summer [10,53], males should increase the nightly foraging time to offset the higher energy costs. Given the multi-faceted threats that insectivores face due to changing resource abundances [54,55] and nutritional availability [56,57], shifting phenologies [58,59], and widespread habitat loss [60], understanding of how energy budgets are shaped when facing varying physiological, ecological, and environmental demands (i.e. reproduction, cold weather, low food availability) and their ultimate links to survival and fitness are crucial.

## 2. Methods

### (a) Study animals

We studied male *N. noctula* from a population in southern Germany (hereafter ‘Konstanz’, 47°39′59.8″N, 9°10′53.6″E). We removed the bats from bat boxes during the day or caught them emerging from a summer bachelor colony in natural tree cavities at dusk with mist nets (Ecotone, Gdynia, Poland) and transported them in individual soft cloth bags to the nearby Max Planck Institute of Animal Behaviour. All bats were adult at the time of capture. We studied male bats just after hibernation in spring (7 April 2020 to 20 April 2020; *n* = 7; ‘spring’ bats) and in early summer 2020 (12 June 2020 to 1 July 2020; *n* = 9; ‘summer’ bats). After transport to the laboratory, we weighed bats with a digital scale ( ±0.01 g; Kern & Sohn, Bahlingen, Germany) and described stage of spermatogenesis based on testicular length ( ±0.01 mm, Mahr IP67 MarCal 16EW). Bats received mealworms and water ad libitum during their natural foraging time at dusk.

### (b) Calibration of heart rate and oxygen consumption in the laboratory

With all bats we performed one or two respirometry experiments, in which we measured oxygen consumption and heart rate. We placed bats individually in respirometry chambers between 21.00 and 23.30 the night prior to the first experiment to acclimate. To measure O_2_ consumption, we used an open-flow pull-through respirometry system with additional humidity control (Sable Systems International, Las Vegas, NV, USA; see [27] for details of the set-up). In short, bats were placed in small airtight plastic containers (volume = 800 ml) equipped with a plastic grid wrapped in mesh which allowed the bats to roost in a natural hanging position while allowing air circulation. Four mass flow systems pulled humidity-controlled air (DG-4) with a constant flow rate of 150 ml/min through each of the four chambers. A subsampler (RM-8) switched between chambers and O_2_, CO_2_ and water vapour pressure (WVP) were analysed with a field metabolic system. We placed the respirometry chambers into a climate-controlled incubator (KB53, Binder GmbH, Tuttlingen, Germany) with a small opening on the lid to mimic the roost or bat box entrance and set the light regime in the room to the natural local circadian rhythm. We calculated individual rates of O_2_ consumption and CO_2_ production using equations 11.7 and 11.8 from Lighton *et al*. [61]. We removed the first 30 s after a channel switch, corrected for drift using a spline fit [62], and phase-matched the raw O_2_ and CO_2_ data. We corrected for water vapour dilution, calculated incurrent and excurrent fractional gas concentrations and dry corrected using equation 8.6 from Lighton *et al*. [61]. We standardized O_2_ consumption with the mean of the body mass before and after experiments and report O_2_ consumption in ml O_2_ g^−1^ h^−1^. To express energy expenditures in kilojoules (kJ), we assumed the caloric equivalent of 1 ml O_2_ as 20.083 J [7].

On one day, all bats were then exposed to a range of temperatures in six increasing 1 h increments (0, 7.5, 15, 22.5, 27.5 and 32.5°C) from 06.00 to 12.00, to investigate if they could also use torpor at higher temperatures (electronic supplementary material, figure S3*c*, table S1.2). With three spring and nine summer bats, we performed an additional experiment for another study [36] in which we simulated natural ambient temperatures over a 12 h period in the laboratory from 06.00 to 18.00 and simultaneously measured the O_2_ consumption and heart rate. To increase sample size, we used data from both experiments if available to calibrate O_2_ consumption and heart rate of each individual at a range of ambient temperatures (electronic supplementary material, figure S3*a*, table S1.1). We held bats in captivity for a maximum of 3 days. We returned them to the box they had been removed from during the day or released them at the capture site in the evening with external heart rate transmitters (see below) and continuously measured their heart rate until the transmitter fell off after a maximum of 7 days without observable negative consequences for the bats.

### (c) Heart rate radio tracking

We attached external heart rate transmitters (*ca* 0.8 g, 5 × 3 × 8 mm; SP2000 HR Sparrow Systems, Fisher, IL, USA; [63,64]) at least 6 h before the laboratory experiment [27]. A detailed description of transmitter attachment can be found in [27]. Each transmitter emits a continuous signal interrupted by cardiac muscle potentials and we recorded heart rate continuously with receivers (AR8000, AOR Ltd, Tokyo, Japan) connected to digital recorders (Tascam DR-05, Los Angeles, CA, USA). In the field, we placed receivers and recorders in a plastic box in close proximity to the bat roost and attached both devices to a portable power station (Beaudens, Kaluojie E-commerce Co. Ltd, Shenzhen, China) to ensure continuous recording. We monitored the number and duration of flights per night and assessed if bats roosted in a group or alone by counting emerging bats at dusk. To record heart rate during flight in spring bats, we used the same tracking equipment and also followed flying bats with an aeroplane (Cessna 172). In summer, heart rates of flying bats were recorded from the ground for a few minutes at the beginning and end of each foraging flight, as the aeroplane was not allowed to fly after 22.00. We used a custom R script to automatically identify the interruptions of the carrier signal by the muscle potentials and calculated heart rate in beats per minute (b.p.m.; [27,33–35]). Automatically analysed files were visually subsampled frequently to validate the filtering method, particularly when variation in heart rate was high. One observer (L.K.) manually counted heartbeats when automated analysis was not possible due to interference or noise.

### (d) Energy content of insects

*Nyctalus noctula* is a generalist insectivore that often feeds on swarming insects. The main insects in their diet belong to the orders Diptera, Lepidoptera, Coleoptera and Trichoptera [40,41,65]. For each order, we chose example species with known caloric content [29,66,67]. We focused on Chironomidae, small Diptera that occur in large swarms in our study area [41,68]; and June beetles, relatively large Coleoptera that are often found in the diet of *N. noctula* [40,41]. From those example species, we calculated the number of insects the bats would need to eat to meet their energetic requirements in both reproductive states. We assumed an assimilation efficiency of 75% [16].

### (e) Data analysis and statistics

We compared testicular length and body mass between spring and summer bats using Welsh’s two-sample *t*-tests. We excluded all recordings before the first flight to exclude the possibility that captivity and handling affected the bats’ behaviour. Only as soon as a bat would fly and choose its own roost, we would assume that the physiology and behaviour would be normal. We defined 3 day-phases: ‘daytime’ as the time between sunrise and emergence from the roost, ‘nighttime’ as the time the bat was in the roost after sunset and before sunrise, excluding flight times, and ‘flight’ as the time the bat was flying. We included only flights from which we had at least 3 min of recordings. In total, we had 11 daytime, 9 nighttime and 13 flight recordings from a total of 7 spring bats and 18 daytime, 18 nighttime and 11 flight recordings from a total of 6 summer bats. Average recording lengths were 738 ± 237 min for daytime, 298 ± 141 min for nighttime and 29 ± 24 min for flight. We calculated mean heart rates per individual and day-phase across different days and compared spring and summer bats using generalized linear mixed models (GLMM, Gamma distribution with log link function, [69]) with bat identification number (batID) as a random effect to account for repeated measures from the same individuals (electronic supplementary material, table S2.1). For pairwise comparisons, we used emmeans (adjustment method Tukey [70]; electronic supplementary material, table S2.2). We defined torpid and resting phases (physiological state) based on visual inspection of the heart rate profiles of each individual and excluded arousal and entry phases [27]. Based on this, we calculated relative time spent torpid and mean torpor heart rates per individual across different days.

To investigate if ambient temperature would affect heart rate during resting or torpor during the day, we used a GLMM (Gamma distribution identity link) with an interaction between ambient temperature and the physiological state (resting or torpor) and batID as a random effect (electronic supplementary material, figure S3*b*, table S3). We obtained hourly air temperature data for Konstanz from the Climate Data Center of the German Weather Service (DWD, https://opendata.dwd.de/climate_environment/CDC).

To predict the energy expenditure of free-ranging bats based on heart rate, we calibrated heart rate with O_2_ consumption using data from the respirometry experiment(s) and the heart rate recordings in the laboratory. For each individual, we calculated a calibration equation using linear regression to obtain the relationship of log(O_2_ consumption) and log(heart rate). Using linear mixed models (LMM) with batID as a random effect, we calculated that the correlation between predicted O_2_ consumption and measured O_2_ consumption was high (*R* ^2^ = 0.90; electronic supplementary material, figure S3*a*, table S1.1). Using these predicted energy expenditures, we followed the same procedure as for the mean heart rate calculations: we calculated mean energy expenditure per individual and day-phase across different days and compared spring and summer bats using GLMMs (Gamma distribution with log link function) with batID as a random effect. For pairwise comparisons, we used emmeans (adjustment method Tukey [70]; electronic supplementary material, tables S2.1 and S2.3). To estimate daytime energy expenditure when spring bats were torpid, we used the previously assigned torpor phases based on heart rate and calculated the mean energy expenditure per individual across different days.

To test whether bats would have the ability to also use torpor at higher temperatures, we used respirometry data collected when bats were exposed to increasing ambient temperatures. All but one summer bat that did not enter torpor were used in the analysis. We fitted GLMMs (Gamma distribution with log link function) to explore variation in response variables (O_2_ consumption, heart rate) based on predictor variables (ambient temperature, season) with individual (batID) as a random factor (electronic supplementary material, figure S3*c*, table S1.2).

For the calculation of individual total daily energy expenditures (DEE), we first calculated for each bat-day the total energy expenditure per minute (l O_2_/min) for the 3 day-phases. We worked with data from a total of 16 bat-days (10 during spring, 6 during summer), from 4 spring and 4 summer bats. We used only bat-days from which gaps in the recordings were between 0 and 35%. To fill these gaps in the recordings, we calculated the mean daytime, nighttime and flight energy expenditure and multiplied this by the length of the gap and added it to the total energy expenditure in each day-phase category. Based on this, we then calculated DEE for each bat-day (in kJ d^−1^) and calculated the total mean DEE for spring and summer bats which we compared using GLMMs (Gamma distribution with log link function; electronic supplementary material, table S4). We compared absolute energy expenditures in each day-phase category in spring and summer using GLMMs (Gamma distribution with log link function) with batID as a random effect. For pairwise comparisons, we used emmeans (adjustment method Tukey [47]; electronic supplementary material, tables S5.1 and S5.2). Based on the total mean DEE for each bat-day, we also calculated the percentage of DEE that the bats used in each of the three day-phases, which we compared using glmmTMB (Beta family logit link, [71]) with batID as a random effect, followed by Tukey HSD tests for pairwise comparison (electronic supplementary material, tables S6.1 and S6.2).

We compared the nightly foraging time of spring and summer bats using LMM with batID as a random effect to account for repeated measures from the same individuals (electronic supplementary material, table S7). For visualization of bat foraging areas, we used GPS fixes from the aeroplane that followed the bats for heart rate telemetry (electronic supplementary material, figure S4). All analyses were performed in R (v. R 4.0.2 [72], RStudio v. 1.1.456 [73]). Data files and code associated with this study are deposited in the Open Research Data Repository of the Max Planck Society ‘Edmond’ [74].

## 3. Results

We tracked free-ranging male *N. noctula* in spring immediately after hibernation and in summer. When roosting, spring males were mostly solitary or shared the roost with a maximum of nine other individuals. All summer males were social and shared the roost with at least 24 other individuals. While body mass did not change between spring (27.2 ± 1.2 g) and summer (27.4 ± 1.8 g; *t*‐test: *t*(12.19) = −0.35; *p* = 0.73), testes length increased by 41% from spring (5.9 ± 1.0 mm) to summer (8.3 ± 1.1 mm; *t*‐test: *t*(13.00) = −4.39; *p* < 0.001), confirming ongoing sperm production. Ambient temperatures ranged from 1.8 to 24.7°C (mean = 13.5 ± 4.2°C) in spring and from 12.7 to 29.6°C (mean = 18.5 ± 3.1°C) in summer.

### (a) Mean daytime heart rate and energy expenditure triple in summer bats compared with spring bats

**Figure 1.**
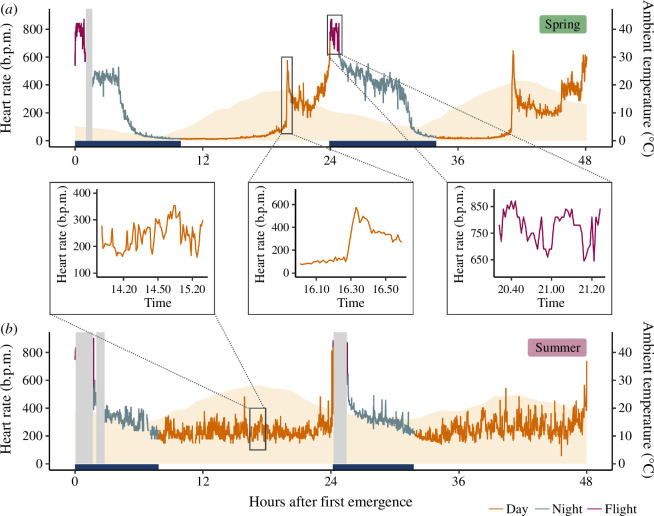
Heart rate tracking of two exemplary bats over 2 days including daytime (orange), nighttime (blue) and flight (purple) recordings. Orange background shading indicates ambient temperature; light grey bars represent missing data; dark blue bars along the *x*-axis denote nighttime. Recordings started at dusk when bats emerged from the roost. (*a*) A spring bat used torpor most of the day with a mid-afternoon arousal on both days (middle insert). (*b*) A summer bat did not use torpor but rested during day roosting with heart rates between 200 and 400 b.p.m. (left insert). Flying heart rates in both bats were between 600 and 900 b.p.m. (right insert).

Mean daytime heart rates (74 ± 39 b.p.m.) and corresponding energy expenditures (0.47 ± 0.26 kJ h^−1^) of spring males were approximately a third of the mean daytime heart rates (252 ± 49 b.p.m.) and energy expenditures (1.42 ± 0.19 kJ h^−1^) of summer males (emmeans *post-hoc* test, both comparisons *p* < 0.001; electronic supplementary material, tables S2.1–S2.3). All spring males used torpor during daytime in the roost (figure 1*a*; electronic supplementary material, figure S1) with strongly decreased and consistently low mean heart rates and thus energy expenditures (26 ± 9 b.p.m.; 0.12 ± 0.07 kJ h^−1^). Occasionally, there were brief arousals during mid-afternoon when temperatures peaked (figure 1, middle insert). On average, spring bats spent 84% of their days and 22% of their nights in torpor. In torpid bats, daytime heart rates were significantly positively correlated with ambient temperatures, while in resting bats, daytime heart rates were significantly negatively correlated with ambient temperatures (*p* < 0.001; electronic supplementary material, figure S3*b*, table S3). Although they experienced similar ambient temperatures as spring bats and had the physiological ability to lower their heart rates at higher temperatures (electronic supplementary material, figure S3*c*, table S1.2), summer bats did not enter torpor at all (electronic supplementary material, figure S2). Instead, their heart rates fluctuated constantly between 200 and 400 b.p.m. (figure 1, left insert). When bats were in the roost during the night, between and after foraging bouts, mean heart rates were significantly elevated compared with daytime heart rates (both comparisons *p* < 0.001) to 360 ± 144 b.p.m. and 350 ± 57 b.p.m., but did not differ from each other (*p* > 0.999; electronic supplementary material, table S2.2). Similarly, corresponding mean nighttime energy expenditures of spring bats (2.54 ± 0.99 kJ h^−1^) and summer bats (2.12 ± 0.65 kJ h^−1^) did not differ from each other (*p* = 0.84) but were elevated compared with daytime energy expenditures (all comparisons *p* < 0.001; electronic supplementary material, table S2.3). Mean heart rates during flight did not differ from each other (spring flight versus summer flight *p* > 0.999) but were significantly elevated to 743 ± 44 b.p.m. in spring and 722 ± 95 b.p.m. in summer compared with daytime heart rates (both comparisons *p* < 0.001; electronic supplementary material, table S2.2). Similarly, resulting flight energy expenditures of spring bats (5.66 ± 0.71 kJ h^−1^) and summer bats (4.43 ± 0.91 kJ h^−1^) were not different from each other but were significantly higher compared with daytime energy expenditure (both comparisons *p* < 0.001; electronic supplementary material, table S2.2; figure 1, right insert, see all pairwise comparisons from the emmeans *post-hoc* tests in electronic supplementary material, tables S2.2 and S2.3).

### (b) Total DEE of summer bats is 42% higher than that of spring bats

**Figure 2.**
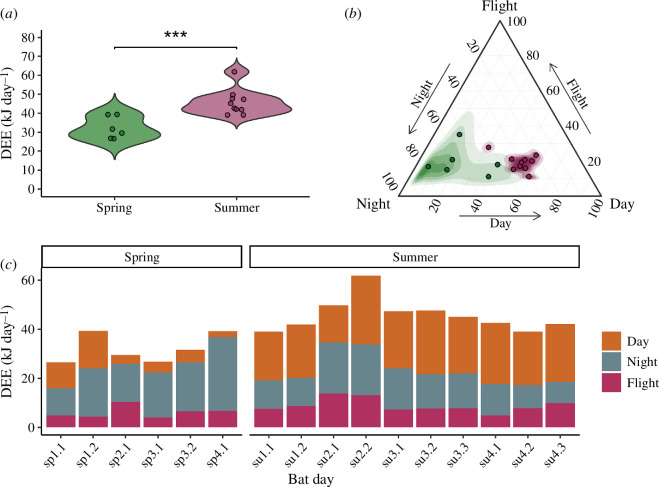
(*a*) Mean DEE in kJ day^−1^ is significantly lower (GLMM, *p* < 0.001) in spring compared with that in summer bats. (*b*) Spring bats (green) spend the highest proportion of their DEE during the night (approximately 60%), while summer bats (purple) spend the highest proportion of their DEE during the day (approximately 50%). Both spring and summer bats allocated approximately 20% of their DEE into flight. (*c*) DEE during daytime (orange), nighttime (blue) and flight (purple) across bat-days in spring (sp) and summer (su). Spring bats have the highest energy expenditure (EE) during the night, while summer bats have the highest EE during the day. Increased flight time in summer elevates flight EE.

We used continuous recordings over 24 h periods including all three day-phases (daytime, nighttime and flight) to calculate total DEE of individuals and to investigate how they budget their time. DEE increased by 42% between spring (32.17 ± 5.84 kJ day^−1^) and summer bats (45.65 ± 6.74 kJ day^−1^; figure 2*a*; electronic supplementary material, table S4). This difference was mainly caused by the drastically increased energy expenditures during daytime in summer compared with spring bats (figure 2*c*, *p* < 0.001; electronic supplementary material, tables S5.1 and S5.2). Summer bats spent around 50% of their total DEE during daytime roosting, which was significantly higher than spring bats which spent only around 22% of their total DEE during daytime roosting (*p* < 0.001; electronic supplementary material, tables S6.1 and S6.2; figure 2*b*). Spring bats spent around 59% of their DEE during nighttime roosting, which was significantly higher compared with summer bats that spent around 30% of their DEE during nighttime roosting (*p* < 0.001; electronic supplementary material, tables S6.1 and S6.2). Despite the relatively small time fractions of each day spent foraging (4 and 8%), around 19% of the total DEE was allocated to flight in both summer and spring bats (figure 2*b*; emmeans *post-hoc* test: spring versus summer flight *p* > 0.999; electronic supplementary material, tables S6.1 and S6.2).

To balance higher summer energy requirements, bats would need to consume 33 June beetles (*Phyllophaga rugosa; Scarabaeidae*; equivalent to 10.0 g fresh insect mass = 37% of the bats’ body mass) or 2572 non-biting midges (*Chironomidae*, 10.6 g fresh insect mass = 39% of the bats’ body mass) each night. Compared with spring, this is 10 more June beetles or approximately 1000 more non-biting midges each night. With the average energy content of a single June beetle (approximately 1.86 kJ), a bat can fuel 20 min of active flight, 38 min of nighttime resting, 59 min of daytime resting or 790 min of torpor in spring.

### (c) Summer bats spend twice as much time foraging as spring bats

**Figure 3.**
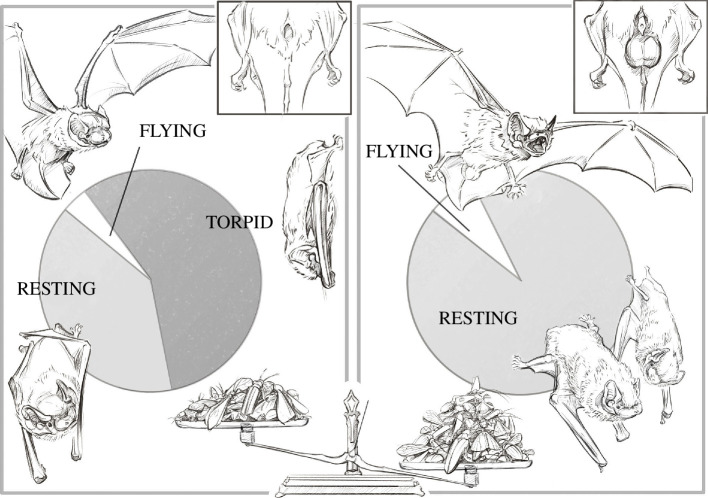
Graphical illustration of the time budget for different physiological states (torpid, resting and flying) in spring bats (left, small testicles) and summer bats (right, large testicles). Spring bats spent 57% of the day torpid, 39% resting, and 4% flying, while summer bats did not enter torpor but spent 92% of the day resting (often social) and 8% of the day flying. To compensate for the 42% higher energy expenditures during spermatogenesis, summer bats need to consume one-third more insects, illustrated by the scale on the bottom. Illustration by Javier Lázaro.

Spring bats spent 96% of the day in the roost (60% during daytime, 36% during nighttime) and only 4% of the day foraging. Distinguishing between physiological states, spring bats spent 57% torpid, 39% resting and 4% flying (figure 3). Summer bats spent 92% of the day resting in the roost (67% during daytime, 25% during nighttime), and 8% of the day flying (figure 3). In absolute numbers, mean flight duration of summer bats (105 ± 38 min) was almost double compared with spring bats (59 ± 15 min; *p* = 0.011; electronic supplementary material, table S7). Spring bats performed one or two flights each night (mean flights per night = 1.4 ± 0.5) with a total nightly flight duration between 43 and 82 min, while summer bats performed one to five flights per night (mean flights per night = 2.5 ± 1.4) with a total nightly flight duration between 35 and 181 min. Spring bats foraged mainly over the shallow water part of the lake, shore and wetland areas and had relatively small foraging areas, to which they returned in consecutive nights (electronic supplementary material, figure S4). Summer bats most likely used similar foraging areas (unpublished data from acoustic monitoring), but detailed tracking with an aeroplane was not possible in summer after 22.00.

## 4. Discussion

As energy is often limited, animals use a suite of behavioural strategies to minimize negative effects on fitness. However, how small free-ranging animals, such as bats, manage their daily energy budgets under these constraints remains largely unknown.

Many species use torpor predominantly when they are not reproductive to avoid potential negative effects on fitness [75–77]. Although reproduction and torpor are widely viewed as mutually exclusive processes [21], many species, including female noctule bats [27], still use torpor occasionally during early [78–80] or late [81–84] pregnancy and lactation without obvious consequences. Torpor has also been observed in male insectivorous bats during sperm production [26,28,84,85]. The free-ranging male bats we monitored using calibrated high-resolution heart rate recorded at roosts and by aeroplane consistently avoided torpor during the summer, causing a dramatic increase of daily energetic costs by 42%. This is in spite of the fact that sperm-producing *N. noctula* can and do use torpor via lowered heart rates extensively in the laboratory (electronic supplementary material, figure S3*c*; [36]). Torpor avoidance in the wild was likely a consequence of ongoing spermatogenesis, as torpor slows down sperm production [15,61,63]. However, we cannot fully exclude other factors, such as self-maintenance and lowering of predation risk, as possible alternative explanations for torpor avoidance [86]. Reproductive individuals reduce the use of energy-saving torpor, as it slows down sperm production [21,26,51]. In spite of higher temperatures, more than 50% of the total daily energy costs during spermatogenesis were generated during daytime roosting, in contrast to only around 22% during spring (figure 2*b*,c). Summer bats offset these additional energetic costs by almost doubling the duration and frequency of foraging bouts during this energetically demanding life-history stage (electronic supplementary material, table S7).

It is important to note the difference between male behaviour observed under laboratory conditions and in the wild similar to conspecific females (electronic supplementary material, figure S3*c*, table S1.2, [27,36]). A laboratory study alone would have revealed the striking ability to uncouple heart rate from body temperature across a range of temperatures but would not have necessarily reflected bats’ behaviour under natural conditions. It will be important in the future to take advantage of increasing availability of technology to take studies to the wild in order to predict how animals will change with ongoing changing conditions.

One possible explanation for the summer torpor avoidance is the suggested relationship between torpor use patterns and the predictability of resources [21]. Species with predictable access to large amounts of food may be strictly homeothermic. Species with unpredictable food, however, may have to use torpor occasionally, at the cost of extending the reproductive period [21]. Our results show that avoiding torpor and maintaining homeothermy increases energetic costs drastically, but speeding up and completing spermatogenesis early, allowing males to be ready for mating sooner and thus increasing mating success, may be more important. In other bat species, insect abundance and foraging duration are positively correlated [30,31]. Given the relatively high nocturnal insect abundance in summer at Lake Constance and in adjacent wetland areas in June [53], increasing foraging duration likely enabled bats to capture enough insect prey. The calculated numbers of June beetles that needed to be consumed to meet DEEs during the reproductive period were comparable to those of *Eptesicus fuscus*, a similar sized North American bat species [66]. In April, when insect abundance is presumably lower and less predictable, bats revert to torpor whenever not foraging and leave the roost mainly briefly during dusk when most swarming insects occur [10]. Bats then immediately return to their roost after exploiting a relatively small foraging area each night (electronic supplementary material, figure S4 [43]). Given the effectiveness of torpor—7 h of torpor requires the same amount of energy as 10 min of flight—it is likely that male noctules would also use torpor in the summer if insect availability was low [28,87]. Future studies from other habitats combined with some measure of food availability will be necessary to understand whether the complete torpor avoidance during the summer we observed is an innate behaviour of male *N. noctula* or due to the particularly productive habitat of Lake Constance [53].

Another compensating mechanism for the higher energetic costs in summer may be the brief formation of social groups that also coincides with sperm production in our and a few species of other temperate-zone bats [88]. While our males were mostly solitary in the spring, they roosted with a minimum of 24 conspecifics in the summer. Similarly, female *N. noctula* prefer warmer roosts for their maternity colonies [89], and social thermoregulation is a common strategy of pregnant and lactating female bats [90–94]. It has been postulated that male bats [88,95] and other animals [96] also profit from social thermoregulation and the possibility of using social information about ephemeral insects during this time [87,97]. This is supported by the fact that males leave these social groups as soon as sperm production is completed and torpor becomes beneficial again [88].

Increasing evidence thus shows that the direct and indirect costs of reproduction are not only high for females but also for males [26,28,29,50,51]. This is particularly interesting in species like *N. noctula*, with sex-biased migration [43,98]. Females are believed to migrate from their wintering habitat to insect-rich areas to buffer the cost of reproduction and raise their offspring under better resource conditions. Meanwhile, males complete spermatogenesis before the females return to be ready for mating. Why only females from our population migrate, and whether only a subset of the males successfully produce high-quality sperm in this polygynous lekking species remains unknown, and future studies looking at migration behaviour, sperm quality and mating success would be valuable.

Aspects of the environment, such as ambient temperatures, may also shape energetic balancing strategies [99,100]. In our study, torpor use at low ambient temperatures was characterized by lower heart rates and consequently lower energy expenditures (electronic supplementary material, figure S3*b*, table S3), and our bats tended to arouse from torpor during midday when ambient temperatures peaked (figure 1*a*, middle insert). These brief arousals may add significantly to DEEs, and with an increasing number of warmer spring days, the number of days on which bats may arouse is also likely to increase. In combination with other changes such as the decreasing availability of well-buffered roosts in old trees or disappearing insects, daily arousals may tip daily energy budgets of bats, causing them to decrease in numbers locally or even at species level. Brief changes in, in this case, heart rate, would remain undetected with more commonly used methods, such as monitoring skin or roost temperatures alone, and we strongly encourage more research collecting high-resolution heart rate data from free-ranging animals. Daily energy consumption increased dramatically, even though resting heart rates decreased at higher ambient temperatures, making the maintenance of homeothermy less costly (electronic supplementary material, figure S3*b*, table S3). Evidently, there is a threshold when torpor through lowered heart rates becomes notably beneficial. Thus, under unfavourable conditions, even reproductive animals revert to using torpor to save energy and bridge times of non-favourable conditions [15,101]. Although this delays the completion of spermatogenesis [51] or the timing of parturition [102], it may increase survival and overall fitness under these circumstances [21].

For many species, optimizing energy budgets is a delicate balancing act between the costs of protracted development, reduced immune function, and predation risk against the benefits of more efficient use of energetic reserves. Flexibility in balancing energy uptake and conservation strategies at all stages of the reproductive cycle increase survival and overall fitness [17]. Understanding how certain energy conservation strategies may be limited at life-history stages and how this is linked to fitness will be critical for predicting how species will respond in a period of rapid environmental change [103]. For example, the sensitivity of bats to disruptions of this fine balance has become apparent through white-nose syndrome, a fungal pathogen that has decimated populations of North American bats by increasing energetic costs during hibernation [104–106]. Recent drastic insect declines [54] may make food availability even more unpredictable and limiting. The adaptive mechanisms of insectivorous bats in response to these drastic declines in insect availability remain unclear. However, as bats consume a considerable quantity of insects and play a crucial role in maintaining ecological balance, this knowledge gap is significant and requires considerable attention and research moving forward.

## Data Availability

Data files and code associated with this study are deposited and accessible in Edmond, the Open Research Data Repository of the Max Planck Society [74].
